# Lighting Up RNA-Cleaving DNAzymes for Biosensing

**DOI:** 10.1155/2012/958683

**Published:** 2012-11-08

**Authors:** Kha Tram, Pushpinder Kanda, Yingfu Li

**Affiliations:** Departments of Biochemistry and Biomedical Sciences and Chemistry and Chemical Biology, Michael G. DeGroote Institute for Infectious Disease Research, McMaster University, 1280 Main Street West, Hamilton, ON, Canada L8S 4K1

## Abstract

The development of the *in vitro* selection technique has allowed the isolation of functional nucleic acids, including catalytic DNA molecules (DNAzymes), from random-sequence pools. The first-ever catalytic DNA obtained by this technique in 1994 is a DNAzyme that cleaves RNA. Since then, many other RNase-like DNAzymes have been reported from multiple *in vitro* selection studies. The discovery of various RNase DNAzymes has in turn stimulated the exploration of these enzymatic species for innovative applications in many different areas of research, including therapeutics, biosensing, and DNA nanotechnology. One particular research topic that has received considerable attention for the past decade is the development of RNase DNAzymes into fluorescent reporters for biosensing applications. This paper provides a concise survey of the most significant achievements within this research topic.

## 1. Introduction

A biosensor is an analytical device composed of two key components: a molecular recognition element (MRE) that seeks a target of interest for binding and a signal transducer that works to translate the target-MRE interaction into a detectable signal. Proteins, particularly antibodies, receptors, and enzymes, have been the traditional choice of MREs in the design of biosensors for many decades. However, nucleic acids that possess a defined function, such as ligand binding and/or catalysis, have emerged as very attractive MREs over the past 20 years [1–3]. These “functional nucleic acids (FNAs)” include DNA and RNA aptamers, ribozymes (RNA-based enzymes), DNAzymes (DNA-based enzymes), and aptazymes (ribozyme-aptamer or DNAzyme-aptamer conjugates in which the catalytic activity of the enzyme domain is regulated by the ligand binding to the aptamer domain). FNAs, and particularly DNA aptamers and DNAzymes, are inherently more stable than proteins, resulting in more robust biosensors that can function for an increased period of time. FNAs can be created to recognize a broad range of targets by a simple test-tube evolution technique known as SELEX (Systematic Evolution of Ligands by EXponential Enrichment) or *in vitro* selection [4–6], a process that is short, does not require the use of animals or cells, and has little restriction on the nature of targets and the choice of experimental conditions. FNAs can be chemically synthesized at a relatively low cost with excellent batch-to-batch consistency. They can be facilely immobilized onto a solid matrix. They are easy to modify with sensing probes to allow the detection by many different methods. Binding of targets to an FNA can be coupled to novel amplification techniques to achieve a high level of signal amplification. Several reviews have been published in recent years that have comprehensively discussed the exploration of FNAs for biosensing applications [1–3]. This review is intended to focus on RNA-cleaving DNAzymes, denoted RNase DNAzymes, as a specific class of FNAs and their use in the development of biosensors that utilize fluorescence as the signal output. Special attention will be given to the most recent developments in this area. 

## 2. Deriving RNA-Cleaving DNAzymes by *In Vitro* Selection 

**Figure 1:**
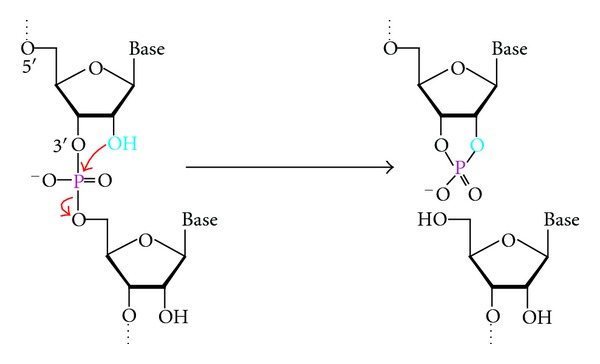
The chemical reaction catalyzed by RNase DNAzymes. These DNAzymes cleave a phosphodiester bond using the 2′-hydroxyl (light blue) as the nucleophile.

Compared to DNA, RNA has significantly reduced chemical stability [7], due to the inherent transesterification reaction between a phosphodiester linkage and the nearby 2′-hydroxyl group (Figure 1). In addition, RNA and DNA are two polymers made of almost identical building blocks, making them prone to interacting through Watson-Crick base pairing. From these two viewpoints, it is not very difficult to make a DNA catalyst that binds an RNA substrate and activates a suitable 2′-hydroxyl group to render its attack on a nearby phosphodiester [8–10]. 

**Figure 2:**
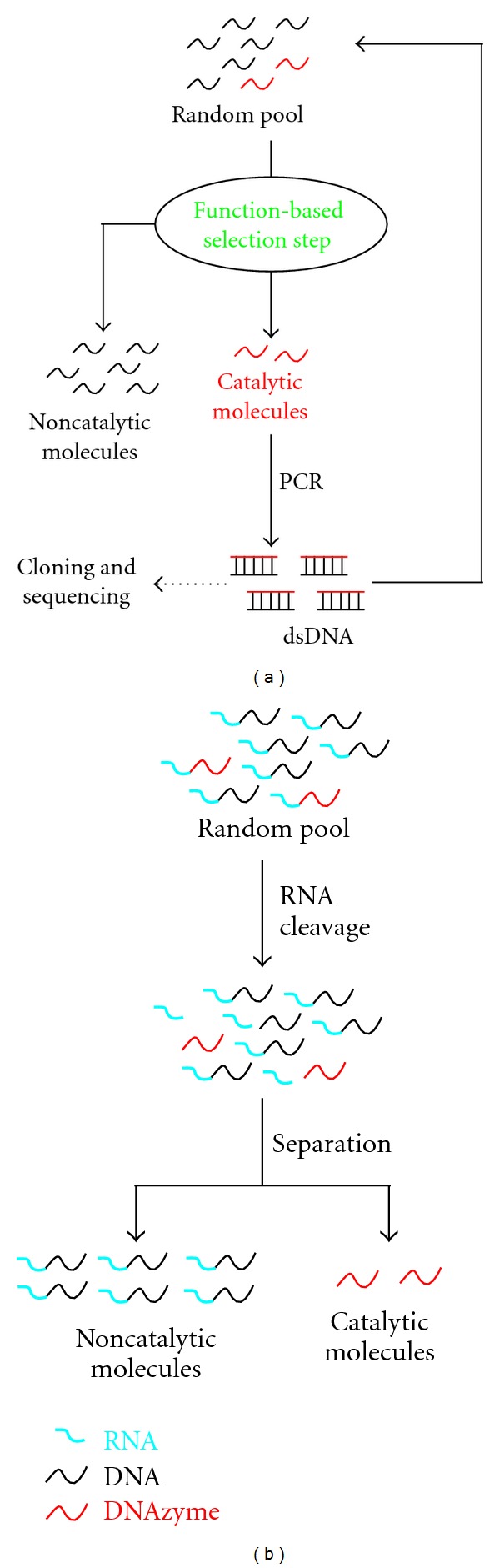
Deriving RNase DNAzymes by *in vitro* selection. (a) Schematic of isolating DNAzymes from a DNA pool. (b) Gel electrophoresis-based method for selecting RNase DNAzymes. For clarity, only the function-based selection step is shown.

All DNAzymes known to date, including RNase DNAzymes, have been obtained from a large, random-sequence DNA library *via in vitro* selection experiments [4–6]. A typical DNAzyme selection (Figure 2(a)) uses a DNA library containing up to 10^16^ different single-stranded DNA molecules. Such a library is chemically synthesized and contains a central random domain flanked by two short constant regions as primer binding sites for polymerase chain reaction (PCR). The library is subjected to a function-based selection step that separates active sequences from inactive ones. The surviving molecules are amplified by PCR and the amplified DNA is used for the next round of selection. The separation-amplification cycle (selection cycle) is repeated for a number of times until the pool exhibits a desirable catalytic activity. This is followed by cloning and sequencing experiments to obtain individual DNAzymes for characterization and application. 

A function-based selection step that can be used to isolate RNase DNAzymes is illustrated in Figure 2(b). The RNA substrate is linked to a DNA pool to isolate *cis*-acting DNAzymes (DNAzymes that cleave an attached RNA sequence). The cleavage makes the active constructs smaller, which travel faster on gel electrophoresis. They are purified, amplified by PCR, and used for the next selection round. This process is repeated until a robust RNA-cleaving activity is observed. Initially, the selected DNAzymes are *cis*-acting, as covalent attachment of the substrate offers an easy way to select for an enzymatic activity; however, *cis*-acting DNAzymes can be made into *trans*-acting DNAzymes (true enzymes) simply by separating the substrate sequence from the DNAzyme.

The first DNAzyme selection, conducted in Gerald Joyce's laboratory in 1994, led to the isolation of an RNase DNAzyme that cleaves a single RNA linkage embedded in a DNA sequence in the presence of Pb^2+^ [11]. The DNAzyme exhibits a cleavage rate of 1 min^−1^, translating into a rate enhancement of 10^5^-fold over the spontaneous cleavage of RNA. Since then, a plethora of *in vitro* selection experiments have been performed to derive diverse RNase DNAzymes with widely different characteristics. The list includes DNAzymes utilizing Mg^2+^ [12], Ca^2+^ [13], Zn^2+^ [14], Mn^2+^ [15], and UO_2_^2+^ [16] as metal-ion cofactors, a cofactorless DNAzyme [17] and DNAzymes that use the amino acid histidine as the cofactor [18]. 

**Figure 3:**
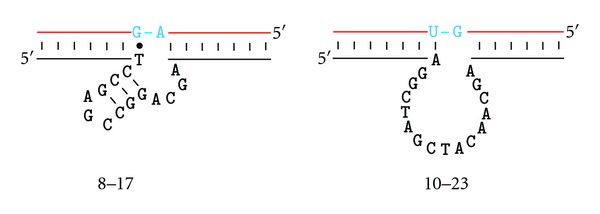
Secondary structures of two best studied RNase DNAzymes: 8–17 and 10–23. The RNA substrate strands are shown in red and the reaction centers are shown in blue. Both 10–23 and 8–17 bind their RNA substrates by two-binding arms made of short Watson-Crick duplexes, which are simplified as line drawings.

The Joyce Laboratory also made an effort of selecting DNAzymes that can cleave an all-RNA substrate under physiological conditions, which led to the discovery of two DNAzymes known as 10–23 and 8–17 (Figure 3) [19]. 10–23 has the ability to cleave any purine-pyrimidine junction within an RNA chain as long as its two-binding arms are properly engineered so that they can form stable duplexes with nucleotides flanking the cleavage junction. 10–23 has been used to target many messenger and viral RNAs for therapeutic applications [8, 9]. The other DNAzyme isolated from this selection, 8–17, has also been isolated in several other *in vitro* selection experiments [13, 14, 20, 21]. Sequence variants of 8–17 are capable of using different metal-ion cofactors, such as Mg^2+^ [19], Ca^2+^ [13], Mn^2+^ [20], and Zn^2+^ [16] as well as cleaving fourteen of the possible sixteen dinucleotide junctions of RNA [20]. 8–17 has been widely studied as biosensors for the detection of metal ions and other ligands [10]; some examples will be discussed below. 

## 3. Dressing Existing RNase DNAzymes with a Fluorescence-Signaling Module

Fluorescence is a powerful technique that has found widespread applications in bioanalysis [22]. Its popularity comes from high detection sensitivity, minimal invasiveness, capability for real-time detection, and the availability of many different fluorophores and related quenchers. The isolation of many RNase DNAzymes has created an opportunity for developing fluorescent biosensors based on such enzymes. 

The most popular way of making a fluorescent sensor out of an RNase DNAzyme is to report the DNAzyme action through the use of a fluorophore/quencher (F/Q) pair. This can be conveniently achieved by a judicious arrangement of F and Q so that they are in close proximity prior to catalysis but separate from each other after catalysis. 

**Figure 4:**
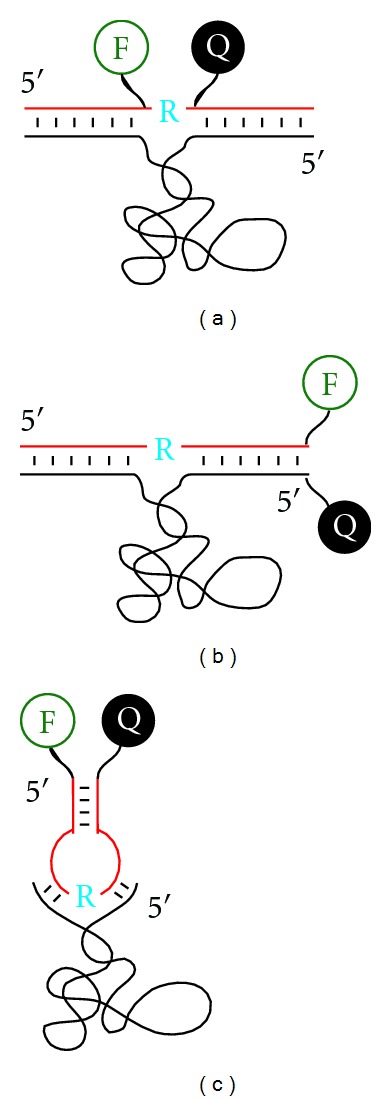
Three common arrangements of fluorophore (F) and quencher (Q) in the design of fluorescent RNase DNAzyme-based biosensors. F and Q are appended to the nucleobases that flank the cleavage site (a), placed at the end of duplex formed between the substrate and DNAzyme strands (b), or located at the end of the hairpin-shaped substrate strand (c). DNAzyme and substrate strands are shown in black and red, respectively. The cleavage site is marked by a blue “R”.

Three types of F/Q arrangements have been commonly adopted: F and Q can be placed on the bases flanking the cleavage site (Figure 4(a)) [23], at the end of a duplex formed between a DNAzyme and its substrate (Figure 4(b)) [24], or at the end of a hairpin-shaped substrate (Figure 4(c)) [25]. 

## 4. Isolating New RNase DNAzymes That Cleave a Fluorogenic Substrate

**Figure 5:**
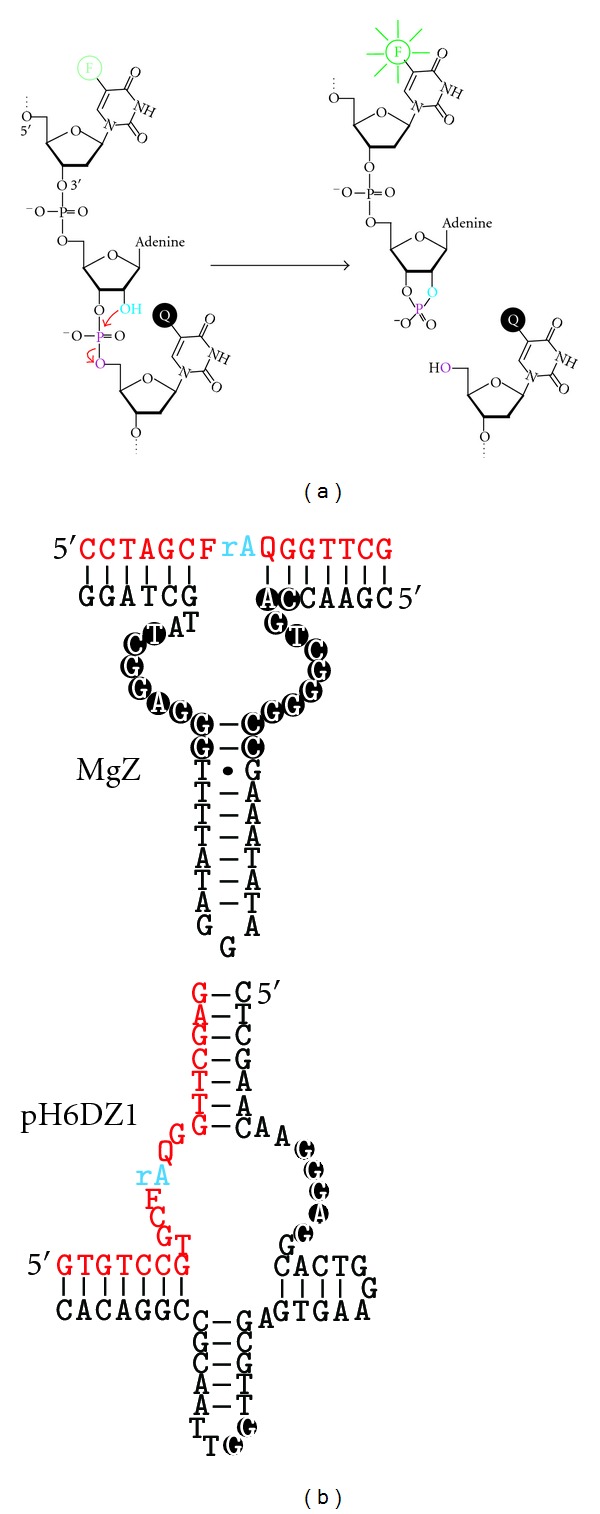
Fluorogenic DNAzymes obtained from random-sequence DNA libraries. (a) The chemical transformation catalyzed by these DNAzymes. These DNAzymes cleave a phosphodiester bond using a nearby 2′-hydroxyl (light blue) as the nucleophile. The special nucleic acid substrate to be cleaved has two features: (1) it contains a single ribonucleotide (riboA) as the cleavage site embedded in a DNA sequence, and (2) the cleavage site is immediately sandwiched between two DNA nucleotides modified with a fluorophore (F; specifically fluorescein) and a quencher (Q; specifically DABCYL). (b) Secondary structures of two representative signaling DNAzymes. rA: adenine ribonucleotide (the cleavage site; shown in blue); F, fluorescein-modified dT; Q, DABCYL-modified dT. Conserved nucleotides are shown in black circles. The substrate strands are shown in red.

In addition to converting existing RNase DNAzymes into fluorescent reporters, novel RNase DNAzymes can also be directly isolated from random-sequence DNA libraries to cleave a substrate predeposited with a F/Q pair. To achieve high levels of fluorescence signal by the dequenching mechanism, F and Q need to be placed as close to the cleavage site as possible. However, doing so can significantly affect the catalytic performance of an existing RNase DNAzyme because the bulky fluorophores and quenchers may prevent the DNAzyme/substrate from achieving optimal structural folding. Isolating RNase DNAzymes directly from a random-sequence library to cleave a premodified substrate would overcome this issue [23]. Our laboratory has carried out several *in vitro* selection experiments to isolate such DNAzymes [26–30]. These DNAzymes cleave a chimeric DNA/RNA substrate containing a single ribonucleotide as the cleavage site that is located between a nucleotide modified with a fluorophore and a nucleotide modified with a quencher (Figure 5(a)). 

Several fluorogenic RNase DNAzymes have been obtained and their catalytic and signaling properties are characterized [31–34]. Most of these DNAzymes are robust catalysts, exhibiting a catalytic rate at or above 1 min^−1^. They can achieve 10- to 30-fold signal enhancements. Some of these DNAzymes have very interesting secondary structures, such as 3-way junction [28, 30], 4-way junction [32], and 5-way junction [29]. The secondary structures of two representative signaling DNAzymes are provided in Figure 5(b).

## 5. Using Fluorescent RNase DNAzymes as Metal Ion Sensors

Certain metal ions, such as Pb^2+^ and Hg^2+^, impose a great threat to human health, and biosensors that have the ability to sensitively and selectively detect such metal ions are highly coveted. DNAzymes typically need divalent metal ions as cofactors to perform efficient catalysis and thus, the catalytic activity of a properly engineered metallo DNAzyme can be exploited to detect metal ions. Significant work has been carried out, mostly in the laboratory of Yi Lu, to develop RNase DNAzymes into sensors for toxic metal ions. Here we will discuss a few examples where the detection was done using fluorescence as the signal output. 

 The first-ever fluorescent DNAzyme-based metal-ion sensor, reported by the Lu group, is for the detection of Pb^2+^ [24]. The DNAzyme employed is a variant of 8–17 that has the robust RNA cleavage activity in the presence of Pb^2+^. The DNAzyme was converted into a fluorescent sensor using the labeling scheme shown in Figure 4(b). This sensor has a detection limit of 10 nM for Pb^2+^ and exhibits an 80-fold selectivity for Pb^2+^ over other metal ions. The same group recently reported another fluorescent Pb^2+^ sensor using the RNase DNAzyme originally selected by the Joyce group in the presence of Pb^2+^ [11]. This sensor was found to be ~40,000 times more specific than the 8–17 based Pb^2+^ sensor [35].

A fluorescent sensor for UO_2_^2+^ was also reported by the Lu group, using an RNase DNAzyme selected for UO_2_^2+^ [16]. This sensor is able to detect UO_2_^2+^ down to 45 pM and exhibits a selectivity of more than one million-fold over other metal ions, making it the best UO_2_^2+^ sensor known to date. 

The same group has also engineered a DNAzyme sensor for Hg^2+^ using an altered version of the uranium-responsive DNAzyme [36]. They replaced a short duplex motif of the DNAzyme with a few T-T mismatches, a DNA element known to bind Hg^2+^. This simple manipulation leads to the deactivation of the DNAzyme. However, when Hg^2+^ is present, the strong binding of Hg^2+^ to the DNA motif reactivates the DNAzyme. This Hg^2+^ sensor exhibits a detection limit of 4 nM, which is below the threshold for toxicity set by the US Environmental Protection Agency. 

## 6. Constructing Fluorescent RNase Aptazymes by Rational Design

To detect molecules other than metal ions, DNAzymes need to carry an aptamer domain as the recognition element. The unified aptamer-DNAzyme systems are often referred to as “allosteric DNAzymes” or simply “aptazymes”. Rational design can be used to engineer RNase aptazymes. This can be done by judiciously integrating an aptamer with an RNase DNAzyme to create an allosteric DNAzyme whose catalytic activity is regulated by the binding of the target analyte to the aptamer domain. A great amount of research work has been carried out to devise various strategies to engineer RNA or DNA aptazymes for biosensing purposes [37–40]. Here we will discuss two strategies published in our own group on linking an ATP-binding DNA aptamer to fluorescently dressed RNase DNAzymes.

**Figure 6:**
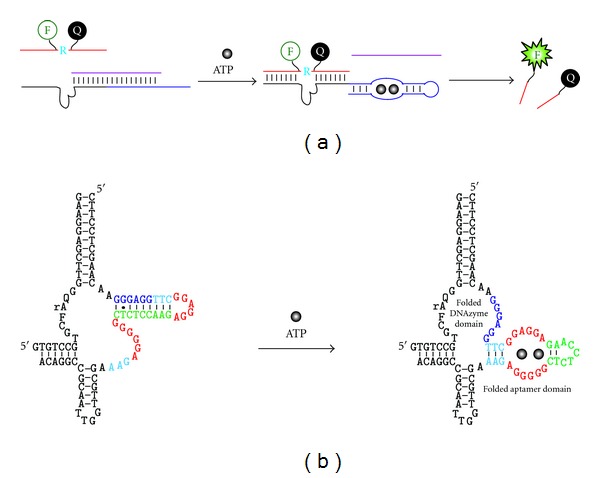
Engineering fluorescent RNase aptazymes by rational design. (a) The use of a regulatory oligonucleotide. The DNAzyme (black line) is joined to an ATP-binding DNA aptamer (blue line) and the combined sequence can form a duplex structure with a regulatory oligonucleotide (purple strand), which prevents the DNAzyme domain from binding the fluorogenic substrate. Binding of ATP to the aptamer domain causes the release of the regulatory oligonucleotide, freeing up the DNAzyme for binding and cleaving the substrate. (b) An ATP-responsive DNAzyme with internal regulatory nucleotides. The DNAzyme is designed from pH6DZ1 and the same ATP-binding aptamer. Nucleotides in the sensor sequence are color-coded for easy tracking of their relative positions in the two alternative structures. In the absence of ATP, the aptamer is designed to form an inhibitory duplex with the catalytic core; in the presence of ATP, the aptamer pulls away from the DNAzyme, which activates the DNAzyme leading to the generation of a fluorescent signal.

The first strategy, illustrated in Figure 6(a), is modeled after the structure-switching aptamer design principle [41]. The strategy uses three separate oligonucleotides: an ATP-binding DNA aptamer linked to an RNase DNAzyme, a regulatory oligonucleotide that binds part of the DNAzyme sequence and part of the aptamer domain, and a fluorogenic substrate [42]. Without ATP, the regulatory oligonucleotide prevents the substrate from binding to the DNAzyme; the binding of ATP to the aptamer results in the release of the regulatory oligonucleotide. The freed DNAzyme can now bind and cleave its fluorogenic RNA substrate, leading to the increase of fluorescence. Two DNAzymes were used for this demonstration: 8–17 and pH7DZ1. 

The second strategy is based on an intramolecular structure-switching mechanism using the same ATP-binding DNA aptamer and pH6DZ1 as the DNAzyme (Figure 6(b)) [43]. The aptamer sequence is inserted into a hairpin motif of pH6DZ1; the sequence of the aptamer is slightly altered so that part of the aptamer can form an intramolecular stem with several catalytically essential nucleotides of pH6DZ1 in the absence of ATP. Upon addition of ATP, the aptamer domain switches from the duplex structure to the complex structure with ATP, which frees up the sequestered nucleotides to create the active structure of the DNAzyme to cleave its fluorogenic substrate. 

## 7. Isolating Fluorescent RNase Aptazymes *via In Vitro* Selection

Although rational design can be used to engineer RNase aptazymes from existing RNase DNAzymes and aptamers, *in vitro* selection can be explored to isolate novel RNase aptazymes from random-sequence pools. Such an approach relies on two selection steps to tune the dependence of the catalytic activity of the DNAzyme on the target of interest: a negative selection step to remove sequences that are catalytic in the absence of target or in the presence of undesired targets; a positive selection step with the target of interest to enrich sequences that are target-specific. 

Our group has used this approach to derive fluorescent RNase DNAzymes that are able to detect bacteria [44, 45]. The goal of the work was to determine whether it was feasible to develop a fluorescent RNase DNAzyme sensor that can detect *Escherichia coli* (*E. coli*), representing the bacteria of interest but does not recognize other bacteria represented by *Bacillus subtilis* (*B. subtilis*). A library of DNA molecules containing a fluorogenic chimeric DNA/RNA substrate was first subjected to a negative selection step with the crude extracellular mixture (CEM) from *B. subtilis*. The cleaved sequences were discarded, and the uncleaved ones were recovered and subjected to a positive selection step with the CEM from *E. coli*. The cleaved sequences from this positive selection step were amplified by PCR and used as the pool for the next round of negative/positive selection. A DNAzyme, named RFD-EC1, was obtained after 20 selection cycles. RFD-EC1 was found to be highly specific for *E. coli* and did not show any activity in the presence of a host of other Gram-negative and Gram-positive bacteria. With the addition of a culturing step, RFD-EC1 is able to detect a single seeding *E. coli* cell. Using the CEM from *E. coli* (which represents a complex mixture of small molecules and proteins) as the target of interest (rather than using a defined biomarker for *E. coli*) allows the selection process to choose a target from the mixture that not only bind strongly to the aptazyme but is also absent from the control bacteria. The key advantage of this method is that it avoids laborious steps to identify and purify a suitable target that is unique only to the target bacteria. 

## 8. Using RNase DNAzymes to Achieve Signal Amplification 

RNase DNAzymes can be neatly incorporated into signal amplification strategies to achieve highly sensitive detection. Here we will discuss three examples.

The first study used the aforementioned Pb^2+^ dependent RNase DNAzyme and an RNA-containing molecular beacon (MB) (as the substrate) to achieve signal amplification. The MB-type substrate provides highly efficient fluorescence quenching; the multiple turnover ability of the DNAzyme—cleaving one MB molecule after another—means that a large signal can be generated from a low concentration of the RNase DNAzyme. The outcome is significantly increased detection sensitivity [46]. 

**Figure 7:**
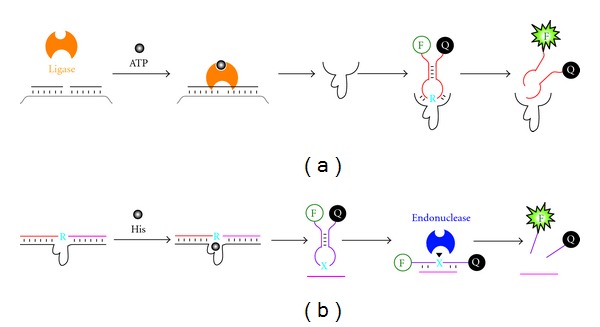
RNase DNAzyme for signal amplification. (a) Ligase/DNAzyme/MB sensor for ATP detection. ATP activates T4 DNA ligase to assemble a whole DNAzyme from two shorter DNA molecules; the DNAzyme cleaves a ribonucleotide (blue “R”) containing molecular beacon (MB) to generate a fluorescence signal. (b) DNAzyme/MB/endonuclease sensors for histidine. Histidine (His) activates the DNAzyme; the cleavage of the substrate by the DNAzyme produces a small DNA fragment (shown as the pink line) that bind an MB to assemble the recognition site (blue “X”) for a nicking endonuclease, which cleaves the MB to generate a fluorescence signal.

The second study cleverly integrated T4 DNA ligase, an RNA-cleaving DNAzyme and again an RNA-containing molecular beacon (MB) to achieve the detection of ATP. Since ATP is a required cofactor for T4 DNA ligase, the presence of ATP in a test sample leads to the activation of the ligase's DNA ligation ability. The activated ligase joins two pieces of DNA to make a functional RNase DNAzyme. The assembled DNAzyme then cleaves the RNA linkage placed within the loop region of the MB to generate a fluorescence signal (Figure 7(a)) [25]. 

The third study employed a histidine-dependent RNA-cleaving DNAzyme, an endonuclease, and an MB to create an amplified sensor for the detection of histidine. When histidine is present, the DNAzyme is activated, leading to the cleavage of an RNA-containing substrate that is designed to bind strongly with the DNAzyme. The cleavage event produces two DNA fragments that can no longer hold onto the DNAzyme strongly. One released fragment goes on to hybridize with the MB creating the double-stranded recognition site for a specific endonuclease, which carries out the cleavage of the MB for fluorescence signal generation (Figure 7(b)) [47]. 

The last two biosensing systems discussed above are encoded with two layers of signal amplification capabilities due to the multiple-turnover nature of both the DNAzyme and the protein enzyme. 

## 9. Conclusions

DNA is best known as the hereditary material for the storage of genetic information in living organisms. However, a great amount of work has been done in the past 20 years that have convincingly shown that DNA is also a versatile polymer from which receptors (DNA aptamers) and catalysts (DNAzymes) can be derived. Since the discovery of the first-ever DNAzyme by the Joyce group in 1994 [11], tremendous progress has been made in DNAzyme research in several aspects, including isolation of many DNAzymes that can collectively catalyze more than a dozen different chemical reactions, demonstration that DNAzymes can achieve large rate enhancements, and application of DNAzymes as chemical and biological tools [9]. One of the highly studied classes of DNAzymes is RNase DNAzyme. This review has sampled some key studies where RNase DNAzymes have been examined for fluorescence-based biosensing applications. 

Several conclusions can be drawn. First, RNase DNAzymes can be made to detect a very broad range of targets, from simple chemical species, such as toxic metal ions and small biological cofactors, to complex biological samples, such as the CEM of *E. coli*. We expect that the list of analytes will expand when this field continues to develop. 

Second, both rational design and *in vitro* selection approaches can be applied to design fluorogenic RNase DNAzymes to detect a specific analyte. Rational design is usually adopted if there is a known aptamer for the analyte. Since aptamers that bind a variety of targets do exist, rational design can still be a fruitful way of turning these aptamers into fluorescent RNase DNAzyme sensors. However, for analytes without a preisolated aptamer, the selection approach becomes an excellent choice. This point has been effectively demonstrated by the Lu group through the creation of a novel uranium sensor [16], as well as by our own group through the development of a novel *E. coli* sensor [44, 45]. We expect that similar approaches will be explored in the future for creating sensors for many other targets.

Third, fluorogenic RNase DNAzyme systems offer a unique way of achieving signal amplification, as illustrated by the two examples discussed earlier where amplified ATP and histidine sensors were engineered. Both examples elegantly use DNA manipulating enzymes (DNA ligase and nicking endonuclease) to produce an RNase DNAzyme (in the case of DNA ligase) or act on the product of an RNase DNAzyme to amplify the signal. Such couplings are made possible simply because DNAzymes are DNA molecules at the first place and can be easily teamed up with DNA manipulating enzymes to do unique things.

The real-time detection capability and excellent chemical stability, combined with the power of *in vitro* selection, will continue to make fluorogenic RNase DNAzyme-based sensors an excellent option for many biosensing applications to come.
